# Association of hypokalemia with an increased risk for medically treated arrhythmias

**DOI:** 10.1371/journal.pone.0217432

**Published:** 2019-06-17

**Authors:** Colin T. Phillips, Junmei Wang, Leo Anthony Celi, Zhengbo Zhang, Mengling Feng

**Affiliations:** 1 Cardiovascular Division, Maine Medical Center, Portland, ME, United States of America; 2 Cardiovascular Division, Beth Israel Deaconess Medical Center, Boston, MA, United States of America; 3 School of Biological Science and Medical Engineering, Beihang University, Beijing, P.R. China; 4 Ping An Health Technology, Beijing, P.R. China; 5 Laboratory for Computational Physiology, MIT Institute for Medical Engineering and Science, Massachusetts Institute of Technology, Cambridge, MA, United States of America; 6 Division of Pulmonary Critical Care and Sleep Medicine, Beth Israel Deaconess Medical Center, Boston, MA, United States of America; 7 Department of Biomedical Engineering, Chinese PLA General Hospital, Beijing, P.R. China; 8 Medical Big Data Center, Chinese PLA General Hospital, Beijing, P.R. China; 9 Saw Swee Hock School of Public Health, National University Health System, National University of Singapore, Singapore, Singapore; University of Pittsburgh, UNITED STATES

## Abstract

**Background:**

Potassium replenishment protocols are often employed across broad patient populations to prevent cardiac arrhythmias. Tailoring potassium thresholds to specific patient populations would reduce unnecessary tasks and cost. The objective of this retrospective cohort study was to determine the threshold at which hypokalemia increases the risk for medically treated arrhythmias in cardiac versus medical and surgical intensive care units.

**Methods:**

Patients captured in the publicly available Philips eICU database were assessed for initiation of either intravenous amiodarone, adenosine, ibutilide, isoproterenol, or lidocaine as a surrogate for a clinically significant arrhythmia. A landmark time-to-event analysis was conducted to investigate the association of serum potassium values and time-marked administration of an antiarrhythmic drug. Analysis was adjusted for comorbidities, the use of vasopressor agents, diuretics, as well as age, gender and severity of illness.

**Results:**

Among 20,665 admissions to cardiac intensive care units, 1,371 (6.6%) were treated with either amiodarone, adenosine, ibutilide, isoproterenol, or lidocaine. For potassium values of ≥3.0<3.5mEq/L, antiarrhythmic treatment occurred at an increased rate compared to a baseline of ≥4.0≤5.0mEq/L (HR 1.23, 95% CI 1.01–1.51; P = 0.04). For admissions to medical and surgical intensive care units, 2,100 of 69,714 patients (3.0%) were treated with either amiodarone, adenosine, ibutilide, isoproterenol, or lidocaine. Potassium values of ≥3.0<3.5mEq/L were also associated with an increased hazard of treatment (HR 1.26, 95% CI 1.09–1.45; P = 0.002). In both cohorts, worsening hypokalemia was associated with an increased risk of antiarrhythmic drug treatment. In neither cohort were there statistically significant differences for serum potassium values of ≥3.5<4.0 and a baseline of ≥4.0≤5.0mEq/L. The proportion of patients initiated on vasopressors or inotropes was over four-fold higher in those treated with one of the antiarrhythmic drugs in both cohorts.

**Conclusions:**

Serum potassium levels <3.5mEq/L were associated with an increased hazard for treatment with specific antiarrhythmic drugs in a large cohort of patients admitted to both a cardiac as well as medical and surgical intensive care units. Potassium thresholds may be individualized further based on risk of relevant outcomes.

## Introduction

Serum hypokalemia is associated with an increased risk of cardiac arrhythmias and sudden cardiac death[1–4]. Potassium homeostasis plays a central role in dysrhythmias, highlighted by seminal observational studies and 2 recent large retrospective cohort studies and a study within the MERLIN-TIMI 36 trial[5–9]. These rigorous studies do not rule out the possibility that hypokalemia is an epiphenomenon and did not measure the serum potassium value at the time of the dysrhythmia but rather at the time of admission, or averaged over the hospitalization [7,8,10].

Expert consensus recommends maintaining serum potassium values of at least 4.0mEq/L in the setting of acute myocardial infarction or congestive heart failure [1,2,11,12]. Potassium replenishment protocols are often applied across the entire hospital population at non-trivial expense and effort based on this evidence from patients admitted with cardiac conditions[3,11,13–15].

The clinical efficacy of replenishing serum potassium in all intensive care unit (ICU) patients is unlikely to be tested in a sufficiently large prospective randomized trial[10,16]. This study helps answer a question that caregivers confront every day in the ICU: at what level does hypokalemia pose an increased risk of an arrhythmia necessitating treatment? The objective of this study is to test the hypothesis that hypokalemia is associated with increased rate of clinically significant arrhythmias in distinct ICU populations as defined by the initiation of either adenosine, ibutilide, isoproterenol, lidocaine or a bolus dose of intravenous amiodarone.

## Methods

### Study design

Longitudinal, multicenter retrospective cohort study from the eICU Collaborative Research Database V1.2 comprised of 335 ICUs across the United States between 2014 and 2015[17]. Patients admitted to either a cardiac or medical and surgical ICU that were older than 18 years with an ICU length-of-stay less than 7 days were included. Patients were excluded if potassium values were above 5mEq/L, or if procainamide, digoxin, or amiodarone were administered without a loading dose suggesting chronic use. This study is compliant with the STrengthening the Reporting of OBservational studies in Epidemiology statement[18]. Use of the Philips eICU database for research and quality improvement is exempt from Institutional Review Board review due to the security schema, for which the reidentification risk was certified as meeting safe harbor standards by an independent privacy expert (Privacert, Cambridge, MA) (Health Insurance Portability and Accountability Act Certification no. 1031219–2).

### Main Exposure/Patients and intervention

Each patient’s ICU admission was divided into 2-hour segments. The maximum serum potassium value within the segment (or value in the closest 2-hour window) was sampled. Serum potassium values were grouped into categories: <3.0, ≥3.0<3.5, ≥3.5<4.0, and a reference of ≥4.0≤5.0mEq/L. A one-sided threshold was chosen given previous evidence demonstrating a U-shaped event curve[7–9,19].

### Outcomes measurement

The primary outcome was time-stamped administration of a loading dose of amiodarone, adenosine, ibutilide, isoproterenol, or lidocaine with loading dose as a surrogate for clinically significant arrhythmias. The rationale for selecting antiarrhythmic drug administration as the outcome is that traditional physiological monitoring including analyzing bedside telemetry has an unacceptable false positive rate of arrhythmias, precluding large scale study[20].

The administration of an antiarrhythmic agent was then matched with the serum potassium associated with that time window. Every serum potassium determination was paired to see whether or not an antiarrhythmic agent was administered within 2 hours of the blood draw. Covariates included age, gender, concurrent intravenous vasopressors and diuretic medications, admission APACHE IV score, and the Charlson Comorbidity Index.

### Statistical analysis

Baseline characteristics were stratified by drug usage. A landmark time-to-event analysis from ICU admission to ICU discharge was conducted to investigate the association of serum potassium values and initiation of an antiarrhythmic drug. The Cox Proportional Hazards model survival analysis at 7 days was used to estimate the adjusted hazard ratios of each group. A multivariate regression model was utilized as a part of the Cox regression with covariates selected on clinical suspicion that they modulated the results. Significance was considered at the p-value < 0.05. Data were analyzed using the R software version 3.4.3.

## Results

**Fig 1:**
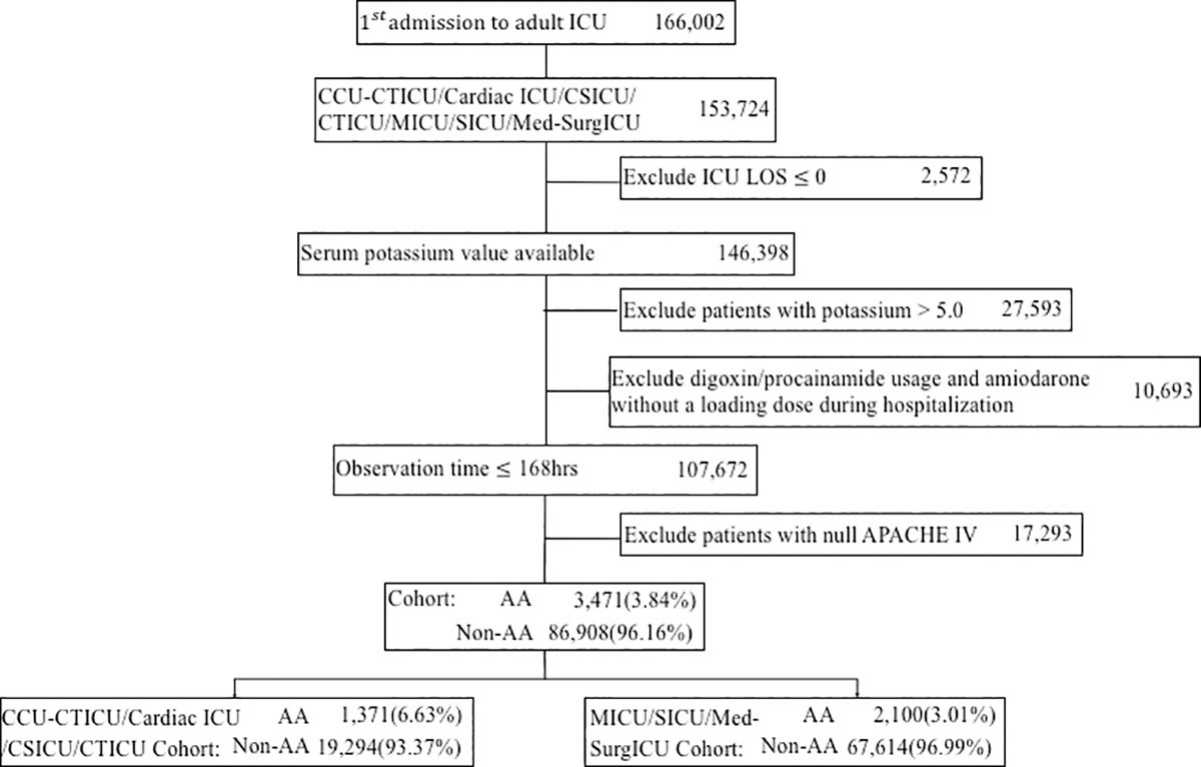
Flow of patients in cardiac ICU and medical and surgical ICU cohorts through the eICU database. AA indicates antiarrhythmic drug treatment; ICU, intensive care unit; LOS, length of stay; CCU, cardiac care unit; CTICU, cardiothoracic ICU; CSICU, cardiac surgery ICU; MICU, medical ICU, SICU, surgical ICU.

Of 20,665 eICU admissions to cardiac ICUs fulfilling the inclusion and exclusion criteria, 1,371 (6.6%) patients were treated with one of the antiarrhythmic drugs of interest (Fig 1). Of 69,714 eICU admissions to either medical or surgical ICUs, 2,100 (3.0%) patients were treated with one of the antiarrhythmic drugs (Fig 1).

In the cardiac ICU cohort and medical and surgical ICU cohorts, the proportion of patients initiated on vasopressors or inotropes within 1 day was over four-fold higher in those treated with one of the antiarrhythmic drugs. Patients treated with antiarrhythmic drugs were older, more often male, and had higher APACHE IV scores and Charlson scores compared to patients not treated with antiarrhythmic drugs (p<0.001 for all comparisons). In both cohorts, there was less of a clear interaction of admission diagnosis and subsequent treatment with an antiarrhythmic drug (Table 1).

**Table 1:** Baseline characteristics of patients included in the analysis according to ICU cohort.

	CICU Cohort			MS ICU Cohort		
Covariate	Non-AA Group	AA Group	p-value	Non-AA Group	AA Group	p-value
	N = 19294 (93.37%)	N = 1371 (6.63%)		N = 67614 (96.99%)	N = 2100 (3.01%)	
Age, years*	65.00 [54.00, 75.00]	68.00 [58.00, 77.00]	<0.001	63.00 [50.00, 75.00]	67.00 [56.00,77.00]	<0.001
**Gender, male %**	10990 57.0	846 61.7	0.001	35110 51.9	1140 54.3	0.035
APACHE IV score*	47.00 [35.00, 61.00]	50.00 [38.00, 64.00]	<0.001	48.00 [35.00, 64.00]	55.00 [40.75,73.00]	<0.001
Charlson score*	3.00 [2.00, 5.00]	4.00 [2.00, 5.00]	<0.001	3.00 [1.00, 5.00]	4.00 [2.00, 6.00]	<0.001
**Vasopressor usage within 1 day, %**	3109 16.1	640 46.7	<0.001	8053 11.9	768 36.6	<0.001
**Inotrope usage within 1 day, %**	1780 9.2	519 37.9	<0.001	2191 3.2	526 25.0	<0.001
**Intubated within 1 day, %**	4545 23.6	621 45.3	<0.001	15020 22.2	648 30.9	<0.001
**History of previous myocardial infarction, %**	2408 12.5	194 14.2	0.079	5090 7.5	246 11.7	<0.001
**History of previous congestive heart failure, %**	2890 15.0	237 17.3	0.024	7883 11.7	319 15.2	<0.001
**History of renal failure, %**	1984 10.3	145 10.6	0.765	6702 9.9	287 13.7	<0.001
**Admission diagnosis of myocardial infarction, %**	2540 13.2	106 7.7	<0.001	2242 3.3	129 6.1	<0.001
**Admission diagnosis of congestive heart failure, %**	812 4.2	22 1.6	<0.001	1957 2.9	54 2.6	0.421
**Admission diagnosis of renal failure, %**	105 0.5	0 0.0	0.011	574 0.8	22 1.0	0.393
**Admission diagnosis of sepsis, %**	1677 8.7	48 3.5	<0.001	10113 15.0	345 16.4	0.067

CICU indicates cardiac intensive care unit; MSICU, combined medical and surgical ICUs.

* Median values and inter-quartile ranges are reported for continuous variables.

**Fig 2:**
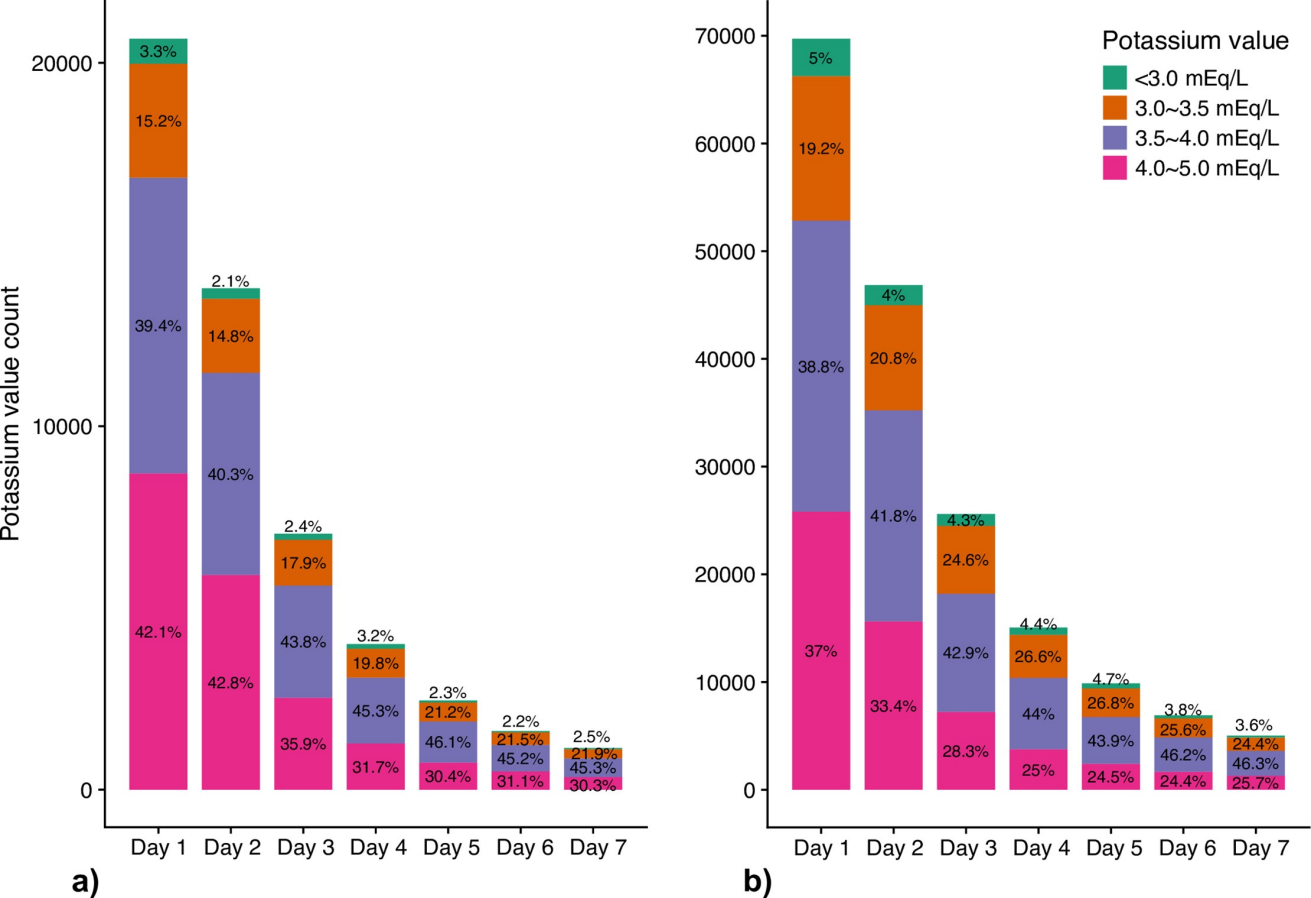
Histogram of potassium values by day in the cardiac ICU (a) and medical and surgical ICU cohorts (b).

Over each of the 7 days analyzed, the proportion of potassium values in each of the categories was similar. For the baseline potassium values of ≥4.0≤5.0mEq/L, between Day 1 and day 7 the proportion ranged from 30 to 42% in the cardiac ICU and 24 to 37% in the medical and surgical ICU cohorts. Likewise, for potassium values between ≥3.0<3.5mEq/L, the range was between 12 and 22% in the cardiac ICU and 19–26% in the medical and surgical ICU cohorts (Fig 2A and 2B).

**Fig 3:**
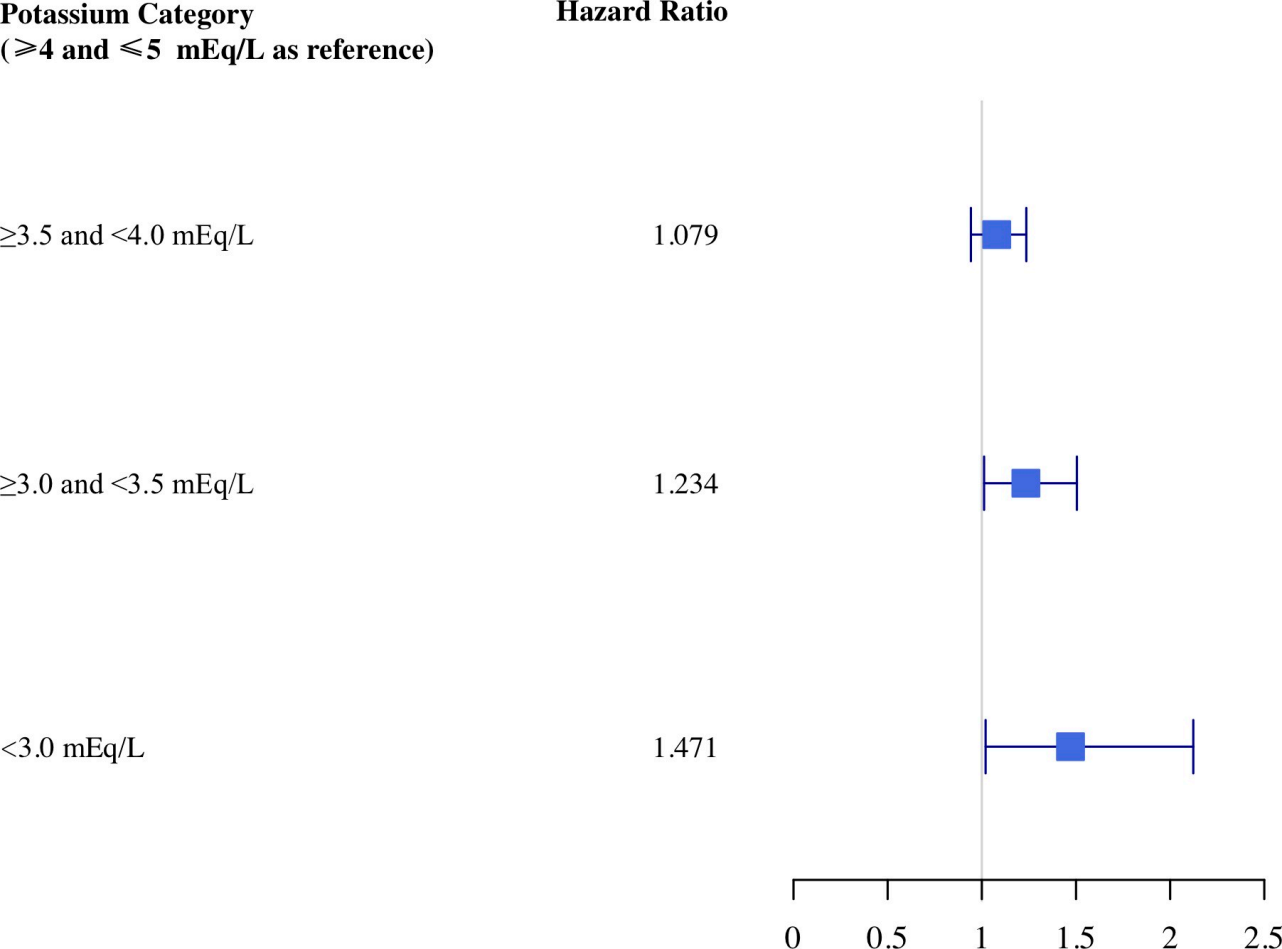
Hazard ratio plot within the first 7 days in the cardiac intensive care unit cohort.

Within the cardiac ICU cohort, patients with serum potassium values ≥3.0<3.5mEq/L had a 23% increased hazard for initiation of either amiodarone, adenosine, ibutilide, isoproterenol, or lidocaine (95% CI 1.01–1.51; P = 0.04). The hazard increased to 47% with serum potassium values <3.0. There was no statistically significant difference in the hazard risk for patients with potassium values ≥3.5<4.0mEq/L and those with potassium values of ≥4.0≤5.0mEq/L (HR 1.08, 95% CI 0.94–1.24; P = 0.27) (Table 2 and Fig 3).

**Table 2:** Cardiac intensive care unit cohort adjusted hazard ratios for initiation of either amiodarone, adenosine, ibutilide, isoproterenol, or lidocaine according to potassium values and other covariates.

Potassium Category (≥4.0≤5.0mEq/L as reference)	Hazard Ratio	95% Confidence Interval Lower Bound	95% Confidence Interval Upper Bound	P-value
≥3.5<4.0	1.079	0.942	1.236	0.273
≥3.0<3.5	1.234	1.012	1.505	0.038
<3.0	1.471	1.020	2.123	0.039

**Fig 4:**
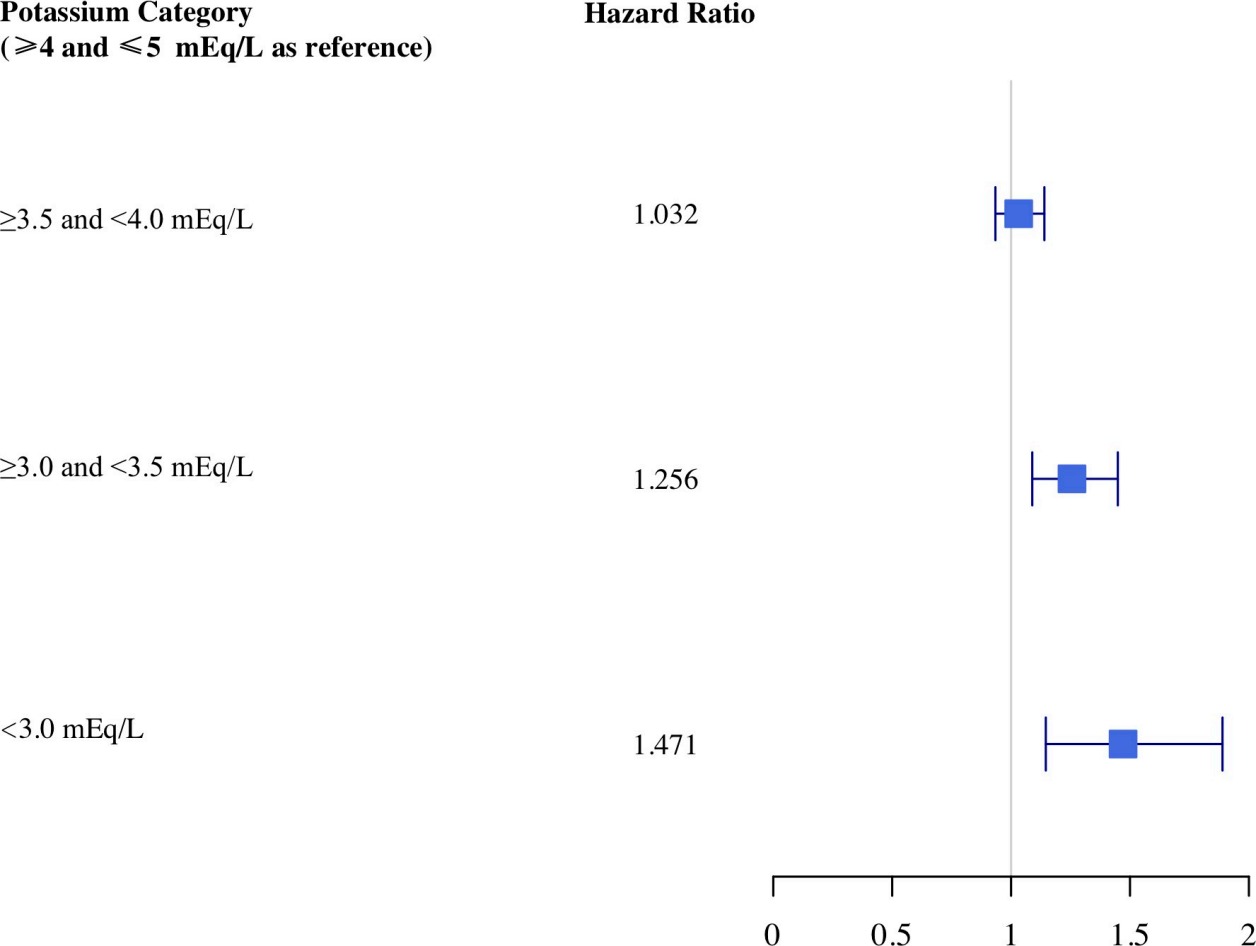
Hazard ratio plot within the first 7 days in the medical and surgical intensive care unit cohort.

Considering the medical and surgical ICU cohort, patients with serum potassium values ≥3.0<3.5mEq/L had a 26% increased hazard of initiation of either amiodarone, adenosine, ibutilide, isoproterenol, or lidocaine (95% CI 1.09–1.45; P = 0.002). The hazard increased to 47% with serum potassium values <3.0mEq/L. There was again no statistically significant difference ≥3.5<4.0mEq/L and the baseline of ≥4.0≤5.0mEq/L (HR 1.03, 95% CI 0.93–1.14; P = 0.54) (Table 3 and Fig 4).

**Table 3:** Medical and surgical intensive care unit cohort adjusted hazard ratios for initiation of either amiodarone, adenosine, ibutilide, isoproterenol, or lidocaine according to potassium values and other covariates.

Potassium Category (≥4.0≤5.0mEq/L as reference)	Hazard Ratio	95% Confidence Interval Lower Bound	95% Confidence Interval Upper Bound	P-value
≥3.5<4.0	1.032	0.934	1.140	0.538
≥3.0<3.5	1.256	1.089	1.449	0.002
<3.0	1.471	1.146	1.889	0.002

## Discussion

Serum potassium levels <3.5mEq/L were associated with an increased hazard for initiation of either amiodarone, adenosine, ibutilide, isoproterenol, or lidocaine compared to a reference range of ≥4.0≤5.0mEq/L in both a cardiac ICU and medical and surgical ICU cohort. There was no statistically significant difference for values between ≥3.5<4.0mEq/L. In both cohorts, the proportion of patients initiated on vasopressors or inotropes within 1 day was over four-fold higher in those treated with one of the antiarrhythmic drugs.

That the threshold for increased events occurred at <3.5mEq/L in the cardiac ICU cohort is counter to expert consensus which recommends a serum potassium of at least 4.0mEq/L in the setting of acute myocardial infarction or congestive heart failure [1,2,11,12]. However, these admission diagnoses only represent 17% of the cardiac ICU cohort studied. The threshold of <3.5mEq/L is consistent with recent investigations demonstrating an increased rate of non-sustained ventricular tachycardia when serum potassium is <3.5mEq/L[9]. When analyzing mean post-admission serum potassium levels in a large cohort of patients admitted with acute myocardial infarction, mortality increased for values <3.5mEq/L and ventricular fibrillation and cardiac arrest when levels <3.0mEq/L[7]. Additionally, when analyzing long-term mortality in patients with acute myocardial infarction, mortality was lowest when serum potassium ranged between 3.5 and 4.0mEq/L[8].

Observational studies using large “real-world” data provide an opportunity to test and refine widely embraced clinical practices such as serum potassium replenishment especially when prospective randomized studies are unlikely to be pursued.

The antiarrhythmic drugs chosen in this analysis have a narrow range of therapeutic uses and therefore act as surrogate markers for specific clinical dysrhythmias [21]. Coupling these drug administration events to time-marked potassium values provides an opportunity to investigate the risk of clinically relevant arrhythmias associated with serum potassium levels. This is particularly true in light of the expected hour-by-hour transcellular potassium fluxes that occur with acidosis, beta-2 receptor activation and stress responses [22,23] This approach can be applied to other databases to tailor the approach of potassium and other electrolyte replenishment protocols. To facilitate replication, all the codes and queries that were used are provided: https://github.com/nus-mornin-lab/PotassiumAA

These current results suggest that lowering serum potassium replenishment thresholds to 3.5mEq/L in both cardiac ICUs and medical and surgical ICUs does not increase the risk of medically treated arrhythmias and would likely reduce the work and resources needed to maintain a potassium of 4.0mEq/L while reducing patient discomfort with administration.

### Limitations

This analysis assumes accurate time-marking of both serum potassium sampling and drug administration. This database did not allow identification of patients treated with electrical cardioversion, although antiarrhythmic agents often are initiated following a cardioversion event. This analysis was also not able to adjust for left ventricular size and function. Finally, serum magnesium was not included as a feature with the assumption of significant co-linearity between serum potassium and magnesium levels[11].

## Conclusions

Hypokalemia <3.5mEq/L was associated with increased risk for medically treated arrhythmias in patients admitted to cardiac and medical and surgical ICUs compared to a reference range of ≥4.0≤5.0mEq/L. In both ICU cohorts, worsening hypokalemia resulted in an increased rate of treatment. The proportion of patients treated with intravenous vasopressors, inotropes, or furosemide was four-fold higher in those treated with antiarrhythmic drugs. This approach can be employed to limit application of thresholds and tailoring potassium replenishment based on admission unit, risk factors, and diagnosis.
